# Demonstration of a Plasmonic Nonlinear Pseudodiode

**DOI:** 10.1021/acs.nanolett.3c00367

**Published:** 2023-04-12

**Authors:** Sergejs Boroviks, Andrei Kiselev, Karim Achouri, Olivier J. F. Martin

**Affiliations:** Nanophotonics and Metrology Laboratory, Swiss Federal Institute of Technology Lausanne (EPFL), 1015 Lausanne, Switzerland

**Keywords:** plasmonics, metasrufaces, bianisotropy, second-harmonic generation, nonlinear optics

## Abstract

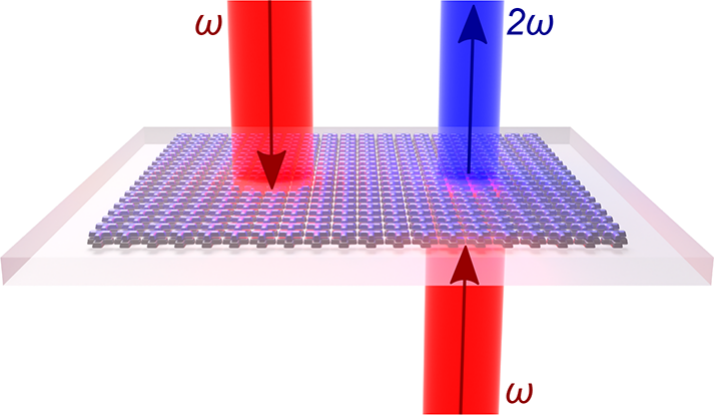

We demonstrate a nonlinear plasmonic metasurface that exhibits strongly asymmetric second-harmonic generation: nonlinear scattering is efficient upon excitation in one direction, and it is substantially suppressed when the excitation direction is reversed, thus enabling a diode-like functionality. A significant (approximately 10 dB) extinction ratio of SHG upon opposite excitations is measured experimentally, and those findings are substantiated with full-wave simulations. This effect is achieved by employing a combination of two commonly used metals—aluminum and silver—producing a material composition asymmetry that results in a bianisotropic response of the system, as confirmed by performing homogenization analysis and extracting an effective susceptibility tensor. Finally, we discuss the implications of our results from the more fundamental perspectives of reciprocity and time-reversal asymmetry.

High-performance nanoscale devices that allow transmission of light only in one direction—optical isolators—remain a long-coveted research objective for optical engineers. This problem is nontrivial due to the fundamental property of electromagnetic waves: in linear time-invariant (LTI) media and in the absence of an external time-odd bias, such as a magnetic field, they propagate reciprocally, i.e. the same way in the forward and backward directions. This property is linked with the time-reversal symmetry of the macroscopic Maxwell equations and can be shown via the Lorentz reciprocity theorem, which specifically applies to LTI media.^1−3^ However, despite recent comprehensive publications on this topic,^1,2,4−6^ there remains a tangible confusion in the community about the difference between true nonreciprocity and the deceptively similar time-reversal asymmetric response. For example, time-invariant and bias-less lossy systems may exhibit contrast upon excitation from opposite directions, but they do not qualify as optical isolators since they possess a symmetric scattering matrix and thus obey Lorentz reciprocity.^7^ Furthermore, in the case of devices based on nonlinear effects, the distinction between true and pseudoisolators is even more intricate. In particular, devices based on Kerr-type nonlinearities^8^ are intrinsically limited by dynamic reciprocity: they can only perform as pseudoisolators, since they do not exhibit unidirectional transmission upon simultaneous excitation from opposite directions.^9,10^ One aim of this letter is to explore possibilities to overcome this limitation and demonstrate how it can be turned into an advantage with an appropriate application.

In that context, photonic metasurfaces—artificial planar materials constituted of subwavelength elements—have been identified as a promising platform for the realization of miniature optical isolators or asymmetric devices.^11^ To this end, let us highlight recent progress in the development of two classes of metasurfaces—nonlinear and bianisotropic metasurfaces. These two classes are particularly relevant to the scope of our work, since combining their features enables the realization of unconventional functionalities, such as the aforementioned nonlinearly induced nonreciprocity,^12−17^ directional harmonic generation,^18−20^ and nonlinear beam shaping.^21,22^

Nonlinear metasurfaces^23−25^ have the potential to replace bulky optical crystals and thus miniaturize nonlinear optical devices. Among other applications, plasmonic metasurfaces have proven to be interesting for second-harmonic generation (SHG),^26−28^ which is a second-order nonlinear optical process in which an excitation wave with frequency ω is converted into a wave with double frequency 2ω.^29^ However, the second-order nonlinear response of plasmonic metals is weak due to their centrosymmetric crystal structure, which is only broken at the surface, giving rise to a nonvanishing surface normal component of the second-order susceptibility tensor χ_⊥⊥⊥_^(2)^. Yet, the overall SHG efficiency remains small due to the reduced interaction volume: essentially, the nonlinear process occurs within the few atomic layers at the metal surface, since the bulk metal is opaque for visible and infrared light and its bulk second-order response is vanishing. Nevertheless, this limitation can be partially overcome by virtue of the field enhancement associated with surface plasmon resonances at metal surfaces. Thus, various SHG enhancement schemes were proposed for plasmonic metasurfaces, based on multipolar resonances,^30−36^ plasmonic lattice resonances,^37,38^ and even light-induced centrosymmetry breaking.^39^

On the other hand, bianisotropic metasurfaces allow engineering the polarization response to realize highly efficient refraction devices through the combination of electric and magnetic effects.^40,41^ The bianisotropic response, which emerges in structures with broken spatial symmetries,^42^ implies that the material effectively acquires magnetic polarization upon excitation with an electric field, and vice versa, electric polarization is produced by a magnetic field. Such a magneto-electric coupling gives rise to the spatial dispersion (i.e., wavevector-dependent response) that enables an excitation-angle-dependent operation.^43^ For example, in lossy systems, it may lead to asymmetric reflection and absorption, which will be discussed further in relation to our work.

In this letter, we demonstrate theoretically and experimentally a plasmonic metasurface that exhibits asymmetric SHG. The operation of the device is conceptually depicted in Figure 1: in contrast to a conventional nonlinear crystal, a second harmonic (SH) is efficiently generated only upon one excitation direction. This, essentially, enables a nonlinear optical pseudodiode functionality, since a nonlinear signal is transmitted only in one direction (though it has to be distinguished from true optical isolators and pseudoisolators, as we discuss in detail further in the letter). Such an asymmetric response imposes a structural asymmetry of the system, and previously proposed theoretical designs with similar functionalities have relied on a geometric asymmetry, which might be difficult to realize experimentally.^44−48^ Here, we take a different route and implement a structural asymmetry through the utilization of two common plasmonic materials—silver (Ag) and aluminum (Al)—in a metasurface and show substantial direction-dependent SHG (up to approximately 16.9 dB in theory and approximately 10 dB in experiment). A major advantage of this two-dimensional design is that such a material asymmetry is relatively easy to implement using standard nanofabrication techniques: e.g., single-exposure electron-beam lithography (EBL).^49^ Furthermore, the combination of plasmonic metals is known to enhance nonlinear processes.^50,51^ To the best of our knowledge, this is the first experimental demonstration of a *plasmonic* metasurface for asymmetric SHG, although we note that in a recent experimental demonstration Kruk et al. utilized a combination of dielectric nonlinear materials for third-harmonic generation.^52^ Additionally, we perform a homogenization analysis of the metasurface to extract effective susceptibilities and reveal the bianiostropic property of our metasurface. Finally, we discuss the fundamental implications of our results in the context of nonreciprocity.

**Figure 1:**
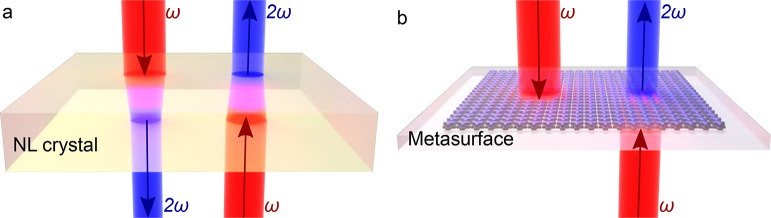
Comparison of conventional and asymmetric SHG: (a) symmetric SHG from a nonlinear (NL) crystal; (b) asymmetric SHG from a nonlinear bianisotropic metasurface.

The building block of the metasurface—the meta-atom—is schematically depicted in Figure 2a. It is comprised of two T-shaped nanostructures made of Al and Ag that are stacked one on top of the other and separated by a thin silicone dioxide (SiO_2_) spacer. These nanostructures are embedded in SiO_2_ and arranged in a square lattice with the period of Λ = 250 nm. Such a periodicity is sufficiently small to avoid diffraction in both linear and nonlinear regimes, as the metasurface is designed for the excitation with the vacuum wavelength of λ_0_ = 800 nm (the effective wavelength in SiO_2_ is ∼537 nm) and SHG at λ_SH_ = 400 nm (∼268 nm in SiO_2_).

**Figure 2:**
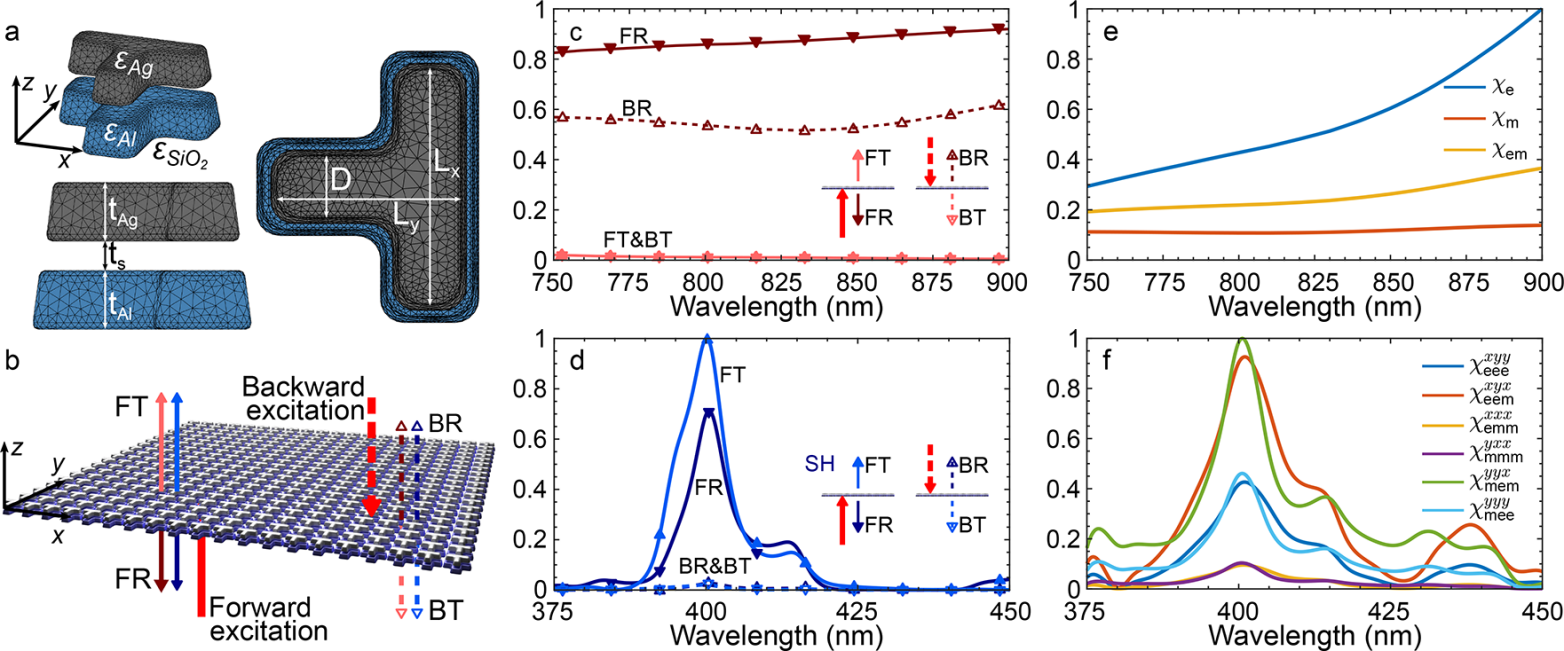
Design and simulated performance of the nonlinear bianisotropic metasurface. (a) Schematics of the considered system: the solid/dashed arrows indicate forward/backward excitations and the arrows colors designate linear (red) and nonlinear (blue) regimes, whereas shades of the colors indicate the transmitted (light) and reflected (dark) waves. Simulated metasurface reflectance and transmittance (c) in the linear regime and (d) at the SH frequency with the same color and line style convention as in (b). Relevant components of the extracted (e) linear and (f) nonlinear effective susceptibility tensors.

As shown in Figure 2b, we consider two different excitation conditions that are indicated with thick red arrows: forward (in the direction along the +*z*-axis, designated with solid lines) and backward (along the −*z*-axis, designated with dashed lines) propagating plane waves that are *x*-polarized. In the linear regime (shown in red), each of the two waves gives rise to transmitted (light shaded lines) and reflected waves (dark shaded lines), which are labeled as forward-excited reflection (FR) and transmission (FT), or backward-excited reflection (BR) and transmission (BT). Additionally, both excitations produce signals at the SH frequency (shown in blue, where the light shade indicates transmission and the dark shade indicates reflection). For the SH waves, we use the same naming convention as the waves produced by linear scattering, and we use the same convention of colors, line styles, and triangular upward/downward arrows throughout the rest of the letter. Also, we note that for the reflected and transmitted waves in the linear regime, we measure the copolarized *x*-component of the electric field, whereas for the SHG waves, the cross-polarized *y*-component is measured, as it is found to be dominant (see Figure S3 in the Supporting Information).

T-shaped meta-atoms provide almost independent control of the spectral positions for the resonances at both the excitation and SH frequencies by varying the lateral dimensions *L*_*x*_ and *L*_*y*_.^53^ As can be seen from Figure S1 in the Supporting Information, for a fixed wavelength, the transmission in the linear regime is tuned by varying *L*_*x*_. In the nonlinear regime, the transmission and reflection are controlled by both *L*_*x*_ and *L*_*y*_. Importantly, for forward excitation, the maximum in SHG transmission coincides with the minimum in linear transmission (compare (a) and (b) in Figure S1 in the Supporting Information). The other geometric parameters *L*_s_, *D*, *t*_Ag_, and *t*_Al_ do not have a strong influence on the resonance wavelength of the fundamental mode; however, they affect the scattering cross-section of the meta-atoms via retardation effects,^54^ which in turn determine the overall transmission and SHG intensity (see Figure S2 in the Supporting Information). The sidewalls of the meta-atom are tilted by 10°, and the edges and corners are rounded with a 5 nm radius to mimic the experimentally fabricated structures, as discussed below.

We select *L*_*x*_ = 135 nm, *L*_*y*_ = 195 nm, *L*_s_ = 25 nm, and *D* = *t*_Ag_ = *t*_Al_ = 50 nm, since these parameters maximize SHG upon forward excitation at the design wavelength, minimize transmission in the linear regime, and result in a sufficiently high extinction ratio of SHG upon forward and backward excitation (see the parametric sweeps in Figure S1 in the Supporting Information). Furthermore, in the *L*_*x*_ and *L*_*y*_ parameter space, the forward-excitation SHG peak is broad, which implies that the metasurface efficiency is weakly sensitive to deviations from the nominal dimensions, thus easing nanofabrication tolerances.

The simulations are performed in two steps using a custom-developed numerical electromagnetic solver based on the surface integral equation.^55,56^ First, the linear fields are computed with a plane wave excitation and periodic boundary conditions. For the SHG simulations, the nonlinear surface polarization *P*_⊥_^(2ω)^ = χ_⊥⊥⊥_^(2)^*E*_⊥_^ω^*E*_⊥_^ω^ is used as a source, where the normal components of the surface fields *E*_⊥_^ω^ are obtained from the linear simulations.

The simulated reflectance and transmittance in the linear and SHG regimes are shown in Figure 2c,d. In the simulations, we use interpolated values ε_Al_ and ε_Ag_ of the experimental permittivity data from McPeak et al.,^57^ and for the permittivity of the background medium we use . Among the noble metals, Ag is known to have the lowest losses at optical frequencies, whereas Al has recently attracted attention as a low-cost alternative plasmonic material.^58−61^ Apart from its low cost, Al is known to have the highest second-order nonlinear susceptibility among the plasmonic materials, in particular its surface normal component χ_⊥⊥⊥_^(2)^;^62^ it also exhibits an interband transition-related absorption peak at 800 nm (see Figure S4 in the Supporting Information).

As shown in Figure 2c, in the linear regime, the transmission *T* for both forward and backward excitations is exactly the same, as imposed by reciprocity. However, the reflection *R* and absorption *A*, which are related to transmission as *A* + *R* = 1 – *T*, depend on the excitation direction, as they are not restricted by reciprocity and depend on the spatial asymmetry of the system. The asymmetric reflection and absorption of the system can be analyzed by considering an isolated meta-atom. As can be seen in Figure S5c,d in the Supporting Information, forward and backward excitations give rise to two distinct electric field distributions. In particular, the electric field concentration in the Al part of the structure is strongly dependent on the excitation direction. Although the response is primarily dipolar for both excitations (see Figure S6a,b in the Supporting Information), this results in asymmetric linear scattering and absorption cross sections, which is a characteristic of *bianisotropic* systems.^17^ In fact, it is the presence of the losses that enables asymmetric scattering when the structure is illuminated from opposite directions, whereas the extinction cross-section remains exactly the same, as imposed by reciprocity.^63^

In turn, the SHG response that is plotted in Figure 2d has an even stronger dependence on the excitation direction: both nonlinear FT and RT are more than 2 orders of magnitude stronger than the BT and BR at 400 nm. A multipolar analysis of an isolated meta-atom (see Figure S6c,d in the Supporting Information) shows that the electric dipolar and quadruplar modes are excited more efficiently at 400 nm upon forward excitation. This is due to the aforementioned different electric-field distributions at the surface of the T-shaped particles, which become the sources for the SHG.

To further elucidate the significance of bianisotropy in such an asymmetric response, we extracted the effective susceptibilities from the simulated electromagnetic fields following the previously documented procedure of metasurface homogenization analysis.^64−66^ Briefly, the expressions for nonlinear susceptibilities are derived from the generalized sheet transition conditions and are calculated using the simulated reflected and transmitted fields upon different excitation conditions at ω and 2ω frequencies.

In Figure 2e,f we plot the extracted effective susceptibility tensor elements that are relevant to the considered excitation conditions. For both linear and nonlinear susceptibilities, the magneto-electric coupling (corresponding to the terms with mixed “e” and “m” subscripts in Figure 2e,f) is non-negligible. The asymmetric response becomes apparent by noting that the induced linear and nonlinear polarizations are given by
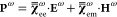
1a
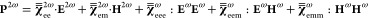
1b

In the linear regime, the non-negligible magneto-electric coupling term χ_me_ results in an asymmetric absorption and reflection. As for the nonlinear effective susceptibility tensors, the dominant components are χ_mem_^*yyx*^ and χ_eem_^*xyx*^, which relate magnetic/electric excitations with electric/magnetic responses along orthogonal directions and result in strongly asymmetric SHG.

To verify experimentally this asymmetric nonlinear response, we fabricated and characterized a metasurface device. Instead of the widespread lift-off process, we employ the ion beam etching (IBE) technique, which enables the fabrication of stratified nanostructures, in particular metal–dielectric composites, with sharper features.^49,67^ The schematic flowchart of the fabrication process is shown in Figure 3a. We use a 150 μm thick D 263 glass wafer (Schott) which is coated with 50 nm thick Al and 25 nm SiO_2_ films using RF sputtering (Pfeiffer SPIDER 600). Next, we deposit a 50 nm thick Ag layer using an e-beam-assisted evaporator (Alliance-Concept EVA 760). The T-shaped pattern arrays are exposed in the hydrogen silsesquioxane (HSQ, XR-1541-006 from DuPont), which is a negative tone e-beam resist, using electron beam lithography (Raith EBPG5000+). The formation of the exposed patterns in the thin films is performed using a low-power argon IBE (Veeco Nexus IBE350, operated at a 300 V acceleration voltage). An important point for this last step is the pulsed IBE operation: 10 s of etching followed by 30 s of cooling to avoid damaging the sample by substrate overheating. The typical overall IBE process time is 160 s, and the etching depth is controlled in situ using a mass spectrometer, which allows real-time monitoring of the etched material composition: the etching process is stopped as soon as the Al flux drops to a minimum. The fabrication results are shown in the scanning electron microscope (SEM) images in Figure 3b–d. The morphology of the fabricated structure can be inspected in Figure 3c: intrinsically, the IBE process results in tilted sidewalls (approximately 10°) and rounded corners and edges. Although such features are typically undesirable, they are not expected to degrade the performance of the metasurface, as these were taken into account in the simulations. In turn, the layered material composition can be well identified in the image acquired with the backscattered electron (BSE) detector in Figure 3d. In the last fabrication step, we cover the metallic nanostructures with a thick SiO_2_ layer (approximately 300 nm), which serves two purposes: it acts as a protective layer preventing degradation of the Al and Ag nanostructures and simplifies the physical conditions by having identical permittivities above and below the metasurface.

**Figure 3:**
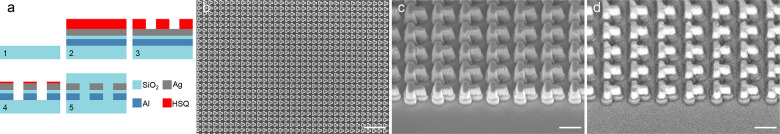
Fabrication of the bimetallic metasurface. (a) Flowchart of the fabrication: (1) initial substrate; (2) Al, SiO_2_, Ag, and HSQ thin film deposition; (3) e-beam exposure; (4) IBE; (5) covering with a thick SiO_2_ film. SEM images of the fabricated structure acquired using different detectors and tilt angles: (b) top view SE (scale bar: 1 μm); (c) 45°-tilted view SE (scale bar: 200 nm); (d) 45°-tilted view BSE (scale bar: 200 nm).

The experimental setup and the results for the optical characterization of the fabricated sample are shown in Figure 4. As an excitation light source, we use a mode-locked Ti:sapphire laser that outputs approximately 120 fs pulses with a central wavelength of 800 nm. The excitation light is weakly focused onto the metasurface with a low-magnification objective (NA = 0.1), which results in a focal spot with a 10 μm full width at half maximum mimicking the plane wave excitation used in the simulations. Additionally, in Figure S7 we show white light linear transmission and reflection spectra upon forward and backward excitations. As expected, FR and BR spectra show asymmetric linear reflection, whereas FT and BT spectra are practically identical. The spectrum of the nonlinear light is shown in Figure 4c. Apart from the characteristic SHG peak at 400 nm, it has a tail at longer wavelengths, which is attributed to nonlinear photoluminescence (NPL).

**Figure 4:**
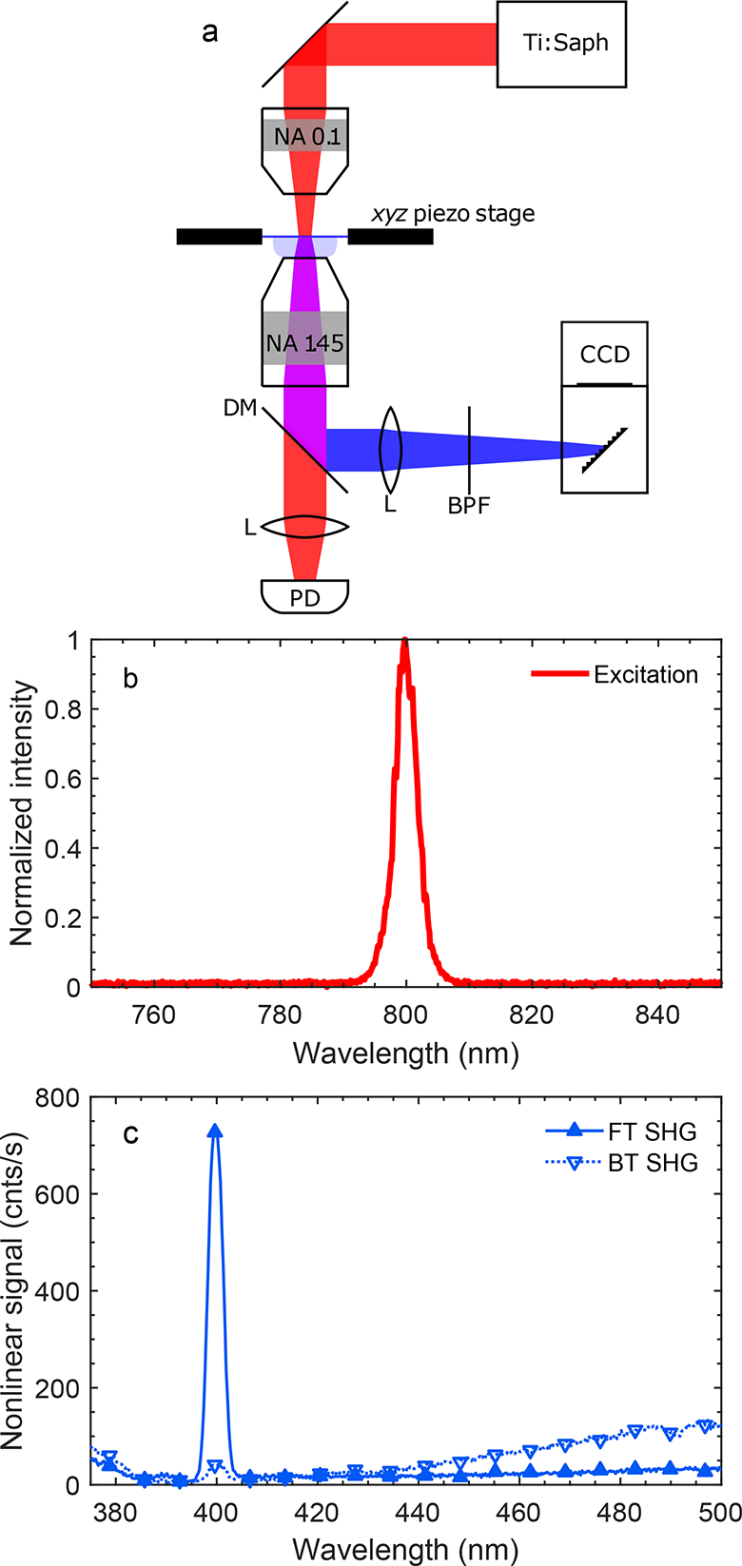
Optical characterization of the metasurface. (a) Measurement setup: a dichroic mirror (DM) in combintation with a short-pass filter (SPF) is used to filter out the excitation light; lenses (L) are used to focus the excitation and nonlinear light onto the photodiode (PD) and the camera (CCD). (b) Excitation spectrum. (c) Nonlinear transmission spectra upon forward and backward excitations.

As an interesting side effect, we note that the NPL signal is substantially larger for BT than for FT. This fact can be explained by the peculiarity of the two-photon absorption mechanism in metals that induces the NPL. As opposed to the coherent nature of two-photon absorption in molecules or dielectrics, in metals it can be regarded as a cascaded process. Specifically, two photons are absorbed sequentially rather than simultaneously.^68−70^ Absorption of the first photon gives rise to an intraband transition in the conduction band and creates a vacancy below the Fermi level. Thus, the second photon results in an interband transition that fills the vacancy in the conduction band and creates one in the valence band. Both of these photon absorption steps are linear but result in an effective nonlinearity. Thus, higher linear absorption upon backward excitation (see Figure S5 and discussion above) results in a higher probability of two-photon absorption and subsequent NPL, which is consistent with our observations.

Such asymmetric behavior is sometimes referred to as “*nonreciprocal SHG*”, both in the metasurface^13,44^ and solid-state physics^71,72^ communities. We share the view that such a nomenclature is improper in the case of SHG, since the concept of nonrecipocity is not well-defined for nonlinear optics.^65,73,74^ For any *N*-port system, the Lorentz reciprocity implies the symmetry of the scattering matrix , where T denotes the transpose operator. In the case of a two-port system like the one considered in this work in the linear regime, the scattering matrix is given by
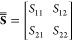
2and reciprocity requires that the transmission coefficients *S*_12_ and *S*_21_ are equal. However, it does not impose any limitations on the reflection coefficients *S*_11_ and *S*_22_. This is true for our system in the linear regime, since the transmissions for forward and backward excitations are equal, while the reflections are asymmetric.

However, in the nonlinear regime, our metasurface can no longer be regarded as a two-port system, since the SH emission represents a distinct electromagnetic mode. Therefore, this system must be at least considered as a four-port system (assuming that higher-order harmonic generation is negligible), represented with the scattering matrix
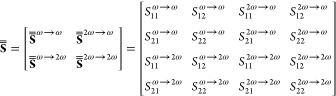
3which describes both linear transmission/reflection at frequencies ω and 2ω, as well as nonlinear processes ω → 2ω and 2ω → ω.

In our experiment, we do not directly probe , where  parameters corresponds to the excitation at SH frequency and generation of a wave at frequency ω. In fact, this process is known as parametric downconversion and it has an extremely low efficiency in comparison with SHG.^29^ Probing this equality, as well as equality of eight other parameters that are flipped by the transpose operation, namely , , ,  and  stand for a true reciprocity test in a four-port system. Instead, within our experiment we show that , which corresponds to an asymmetric nonlinear scattering process that is reciprocal. Yet, a rigorous probing of reciprocity in a nonlinear system would require sophisticated experiments that involve simultaneous excitation with the two waves at frequencies ω and 2ω and precise control over their amplitude and phase.^73^ Nevertheless, we assert that our device essentially functions as a nonlinear optical pseudodiode, allowing efficient excitation of SHG only upon one excitation direction and thus enabling unidirectional SH signal transmission, which is a desired functionality for various signal processing applications.^75^

In summary, we have demonstrated that strongly asymmetric SHG can be achieved in a plasmonic metasurface that is comprised of two common plasmonic metals—aluminum and silver. Our approach in creating structural asymmetry via the material contrast allows us to engineer spatial dispersion, which is required to achieve the asymmetric nonlinear response. The tailored designed meta-atoms produce a dependence on the excitation direction, with an extinction ratio of approximately 16.9 dB in theory and approximately 10 dB in the experiment. We anticipate that our findings can pave the way for further developments in the field of nanoscale bianisotropic and nonreciprocal devices, as well as inspire novel nonlinear plasmonic devices with unrivaled functionalities.

## Supporting Information Available

Additional results of the numerical simulations, including control over the linear and SH transmission via variation of the geometrical parameters of the meta-atom, SH electric field polarization, permittivity data of the used materials, and scattering, absorption, and multipole analysis of the isolated meta-atom (PDF)
