# Supplementary material for: Demonstration of a Plasmonic Nonlinear Pseudodiode
Source: Nano Lett. 2023 Apr 12;23(8):3362–8. doi: 10.1021/acs.nanolett.3c00367 (PMC10141562; doi:10.1021/acs.nanolett.3c00367)
Supplement: Supplementary file 1 — nl3c00367_si_001.pdf [file nl3c00367_si_001.pdf]

Supporting Information for:

Demonstration of a plasmonic nonlinear pseudo-diode

Sergejs Boroviks,* Andrei Kiselev, Karim Achouri, and Olivier J.F. Martin*

Nanophotonics and Metrology Laboratory, Swiss Federal Institute of Technology Lausanne (EPFL), Lausanne, Switzerland

E-mail: sergejs.boroviks@epfl.ch; olivier.martin@epfl.ch

References

[S1] McPeak, K. M.; Jayanti, S. V.; Kress, S. J. P.; Meyer, S.; Iotti, S.; Rossinelli, A.; Norris, D. J. Plasmonic Films Can Easily Be Better: Rules and Recipes. *ACS Photonics* **2015**, *2*, 326–333.

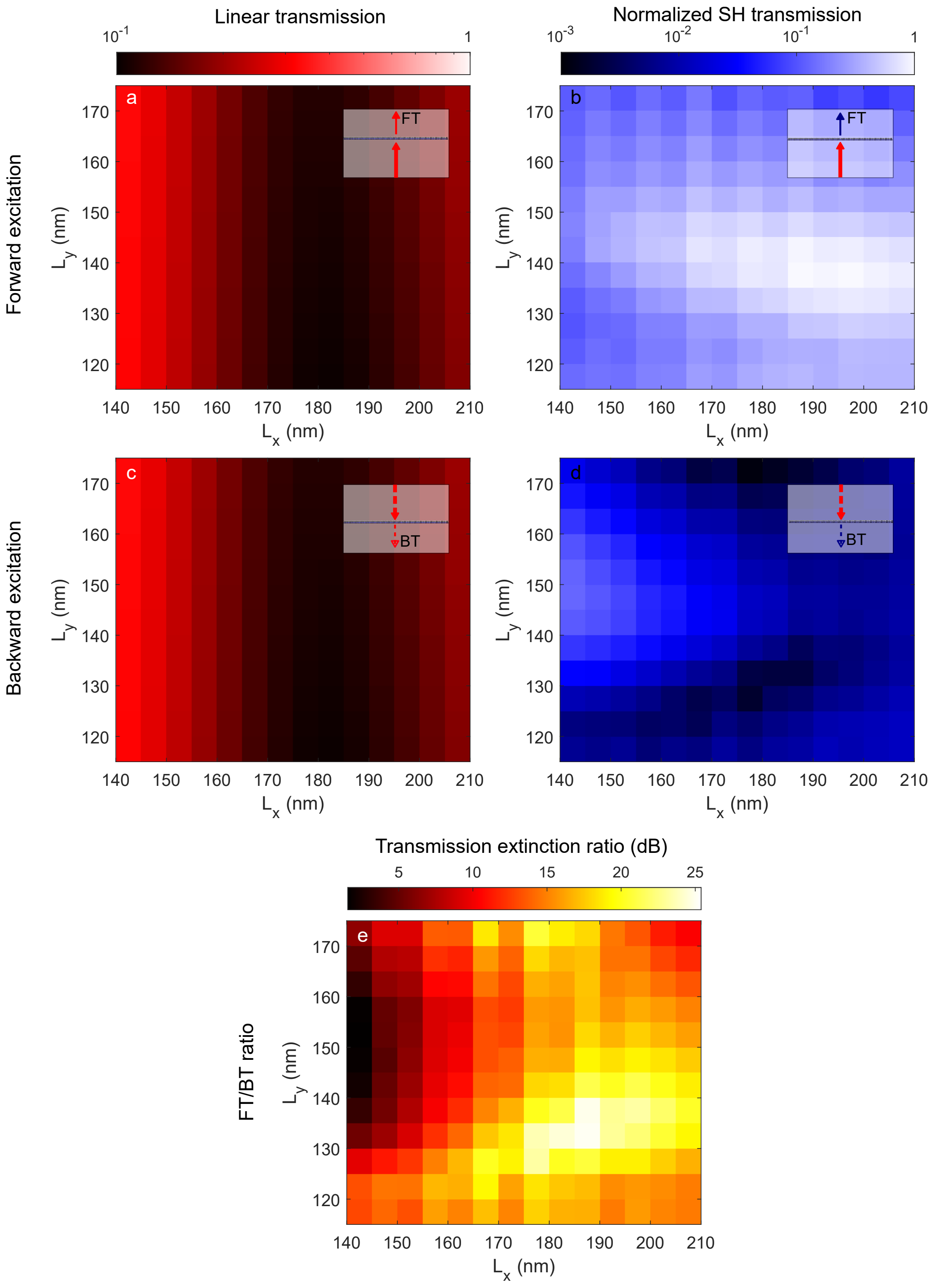

Figure S1: Control over linear and SH transmission via L_x and L_y geometrical parameters at excitation wavelength $\lambda_0 = 800\,\text{nm}$. Other parameters are fixed: $D = t_{\text{Ag}} = t_{\text{Al}} = 50\,\text{nm}$ and $L_s = 25\,\text{nm}$. Simulated (a) linear and (b) SH transmission upon forward excitation; (c) linear and (d) SH transmission upon backward excitation. (e) Forward/backward-excitation SH transmission extinction ratio.

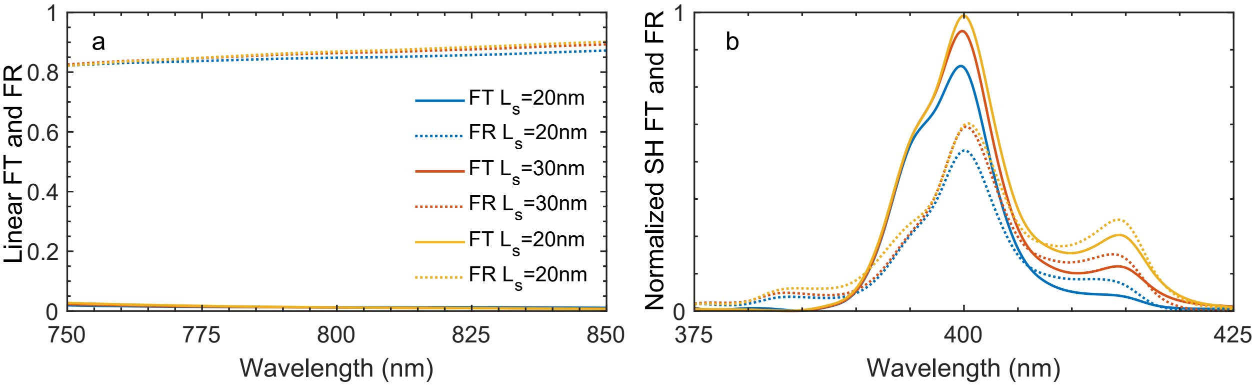

Figure S2: Simulated dependence of the linear and SH transmission and reflection upon forward excitaiton (FT and FR) on the geometrical parameters (a) Linear and (b) SH. Other geometrical parameters are fixed: L_x =135 nm, L_y =195 nm and $D = t_{\mathrm{Ag}} = t_{\mathrm{Al}} = 50$ nm.

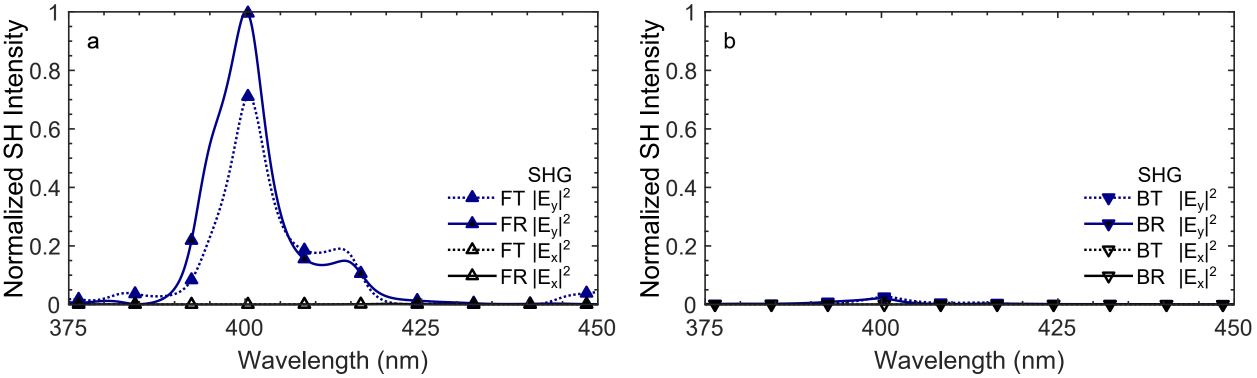

Figure S3: Electric field components extracted from the simulations of the reflected and transmitted SH waves. In both cases, (a) forward excitation (FE) and (b) backward excitation (BE) the $E_y^{2\omega}$ component (that is orthogonal to the excitation field E_x^{ω}) is dominant.

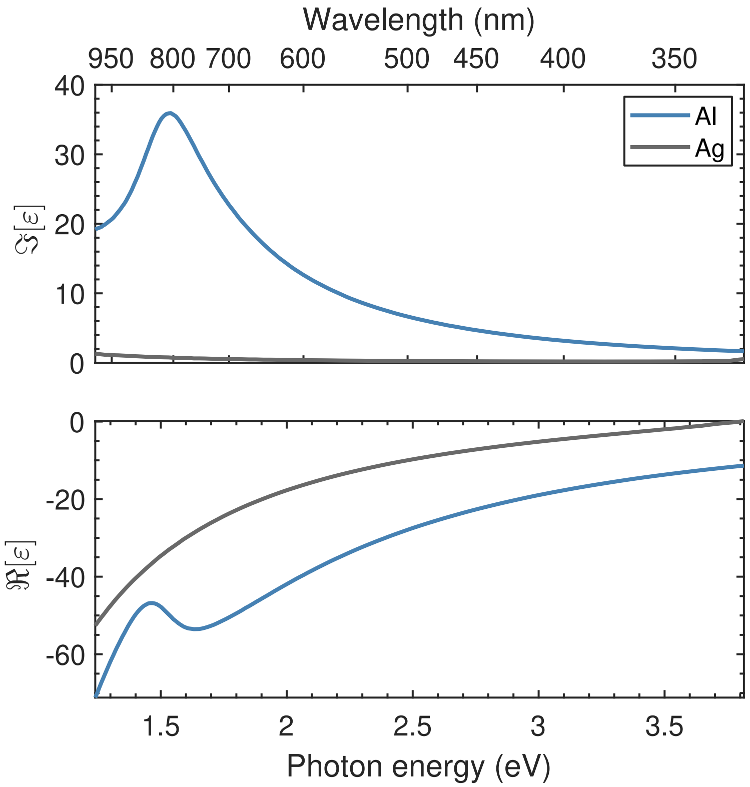

Figure S4: Dielectric permittivity of Al (blue lines) and Ag (gray lines) used in the simulations: real (bottom panel) and imaginary (top panel) parts of the interpolated experimental data from ref.[S1]

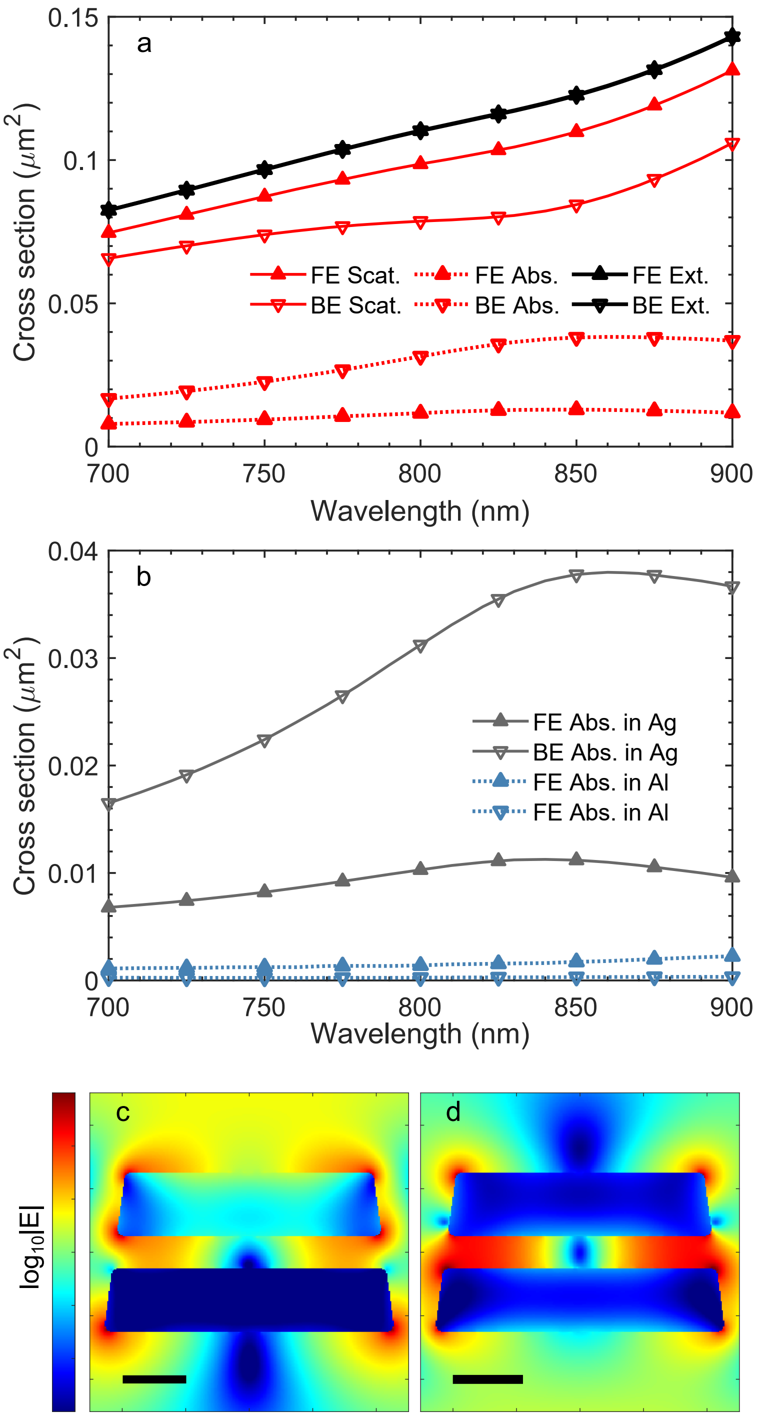

Figure S5: Simulations of isolated meta-atom linear scattering and absorption. (a) Scattering (red solid lines), absorption (red dotted lines) and extinction (black-solid lines) cross-sections upon forward excitation (upward triangles) and backward excitation (downward triangles); (b) absoption in Ag (gray solid lines) and Al (blue dotted lines) domains upon forward excitation (upward triangles) and backward excitation (downward triangles). Pseudo-color images of the normalized magnitude of the electric field distribution upon (c) forward and (d) backward excitations (logarithmic scale; scale bar: 50 µm).

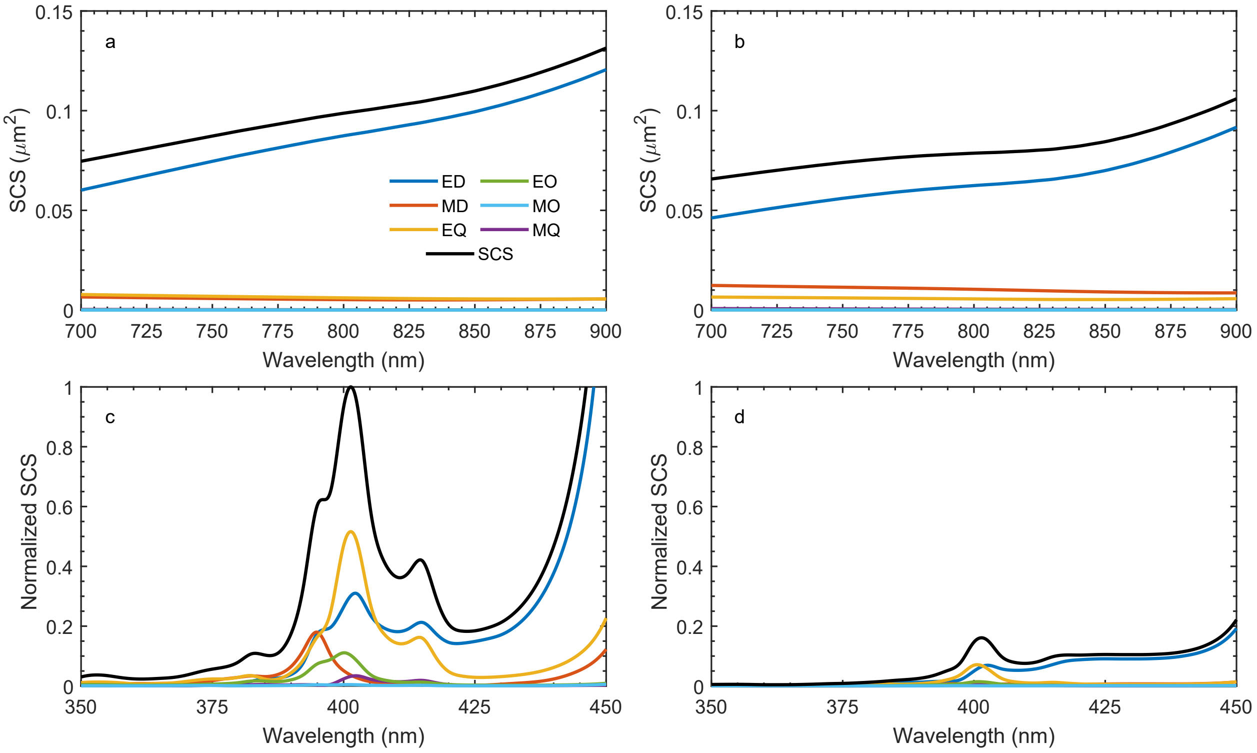

Figure S6: Isolated meta-atom multipole analysis (simulations). Vector spherical harmonic decomposition of (a) linear scattering upon forward excitation; (b) linear scattering upon backward excitation; (c) SHG scattering upon forward excitation; (d) SHG scattering upon backward excitation.

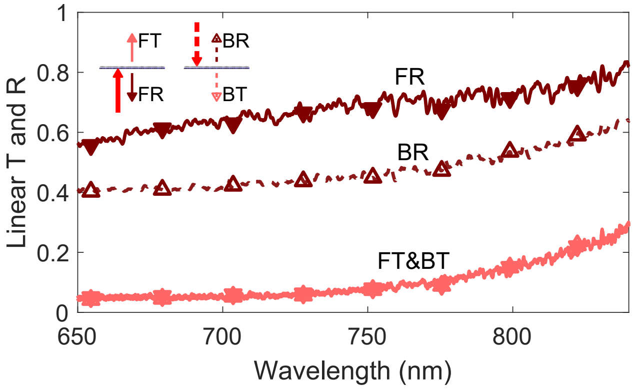

Figure S7: Experimental measurements of the linear transmission and reflection spectra upon forward (FT and FR) and backward (BT and BR) excitations, acquired under white light illumination (standard halogen lamp) with a 50:50 beamsplitter instead of a dichroic mirror.
